# Patent iliolumbar artery increase no risk of type II endoleaks after endovascular abdominal aortic aneurysm: a case-control study

**DOI:** 10.3389/fcvm.2023.1210248

**Published:** 2023-08-11

**Authors:** Guo Xin Chen, Dan Liu, Chengxin Weng, Chuwen Chen, Jianghong Wan, Jichun Zhao, Ding Yuan, Bin Huang, Tiehao Wang

**Affiliations:** ^1^ Department of Vascular Surgery, West China Hospital, Sichuan University, Chengdu, China; ^2^ Department of Outpatient, West China Hospital, Sichuan University, Chengdu, China

**Keywords:** abdominal aortic aneurysm, endovascular aortic repair, type 2 endoleaks, iliolumbar artery, risk factors

## Abstract

**Objective:**

The aims of the present study were to explore the risk factors for type 2 endoleaks (T2ELs) after endovascular aneurysm repair (EVAR) and the association between T2ELs and the iliolumbar artery.

**Materials and methods:**

A single-center, retrospective case–control study in West China Hospital was conducted among patients with infrarenal abdominal aortic aneurysm (AAA) who underwent EVAR between June 2010 and June 2019. The associations of patient characteristics, anatomical factors, internal iliac artery embolization, and ILA with the primary outcome were analyzed. The secondary objective was to analyze survival and reintervention between the T2EL group and the non-T2EL group. Kaplan–Meier survival, propensity matching analysis and multivariate logistic regression analysis were used.

**Results:**

A total of 603 patients were included. The median follow-up was 51 months (range 5.0–106.0). There was a significant difference in the diameter of the lumbar artery (LA), middle sacral artery (MSA) and inferior mesentery artery (IMA), proportion of thrombus and LA numbers. The univariate analysis showed that T2ELs were more likely to develop more thrombus in aneurysm cavity (OR = 0.294, *p* = 0.012), larger MSA (OR = 1.284, *p* = 0.04), LA (OR = 1.520, *p* = 0.015), IMA (OR = 1.056, *p* < 0.001) and more LAs (OR = 1.390, *p* = 0.019). The multivariate analysis showed that the number of LAs (HR: 1.349, 95% CI: 1.140–1.595, *p* < .001) and the diameter of the IMA (HR: 1.328, 95% CI: 1.078–1.636, *p* = 0.008) were significantly associated with T2ELs. There were no new findings from the propensity score matching. The reintervention-free survival rates were significantly different between the two groups (*p* = 0.048). Overall survival and AAA-related death rates were not different between the two group. This was consistent with the PSM analysis.

**Conclusion:**

The iliolumbar artery and the different internal iliac artery interventions may not increase the incidence of T2ELs. But the numbers of LAs and IMA diameter were independent risk factors for T2Els. T2ELs was associated with the reintervention but did not affect long-term survival or increase aneurysm-related mortality after EVAR.

## Introduction

Abdominal aortic aneurysm (AAA) is defined as a disease with an abdominal aortic diameter of more than 3 cm or 50% greater than the normal aortic diameter (1, 2). Although endovascular aortic aneurysm repair (EVAR) has become the first choice of treatment because of its advantages of less trauma, faster recovery and lower perioperative mortality, several studies have shown that the reintervention rate of EVAR is higher than that of OSR (3–5). Endoleaks, an important cause of reintervention, is a common and unique complication of EVAR and occurs in approximately 1/3 of postoperative patients (6). Type II endoleaks (T2ELs) are caused by retrograde blood flow from the side branches of the abdominal aorta entering the aneurysm sac after excluding the aneurysm, and they are the most common type of endoleaks, with an incidence rate between 8% and 44% (7–9).

The treatment methods for T2ELs include trans-lumbar direct embolization of the aneurysm sac, embolization of the aortic branches through the superior mesenteric artery or lumbar arteries, trans-cavity embolization, and open or laparoscopic clipping of side branches (10). Postoperative reintervention of the T2EL is challenging, while intervening in the anatomical risk factors seems to be more advantageous intraoperatively. Abdominal aortic collateral artery embolization can reduce the incidence of T2ELs and the reintervention rate (11–18) and promote the reduction of aneurysms after EVAR (12, 13, 16–18), with a lower incidence of complications (12). The high anatomical risk factors for T2ELs include patent IMA and LA (16, 19, 20). In addition, the incidence of T2ELs was also associated with the internal iliac artery ranging from 0 to 3.8% (21), and some investigators believe it was related to the iliolumbar artery (21, 22). The iliolumbar artery and lumbar artery are connected through collateral circulation and can communicate with the fourth lumbar artery (23–25). However, no studies have investigated the association between the iliolumbar artery and T2ELs after EVAR. The purpose of this study was to investigate the relationship between the iliolumbar artery and T2ELs after EVAR.

## Method

### Study design

This was a single-center, retrospective case–control study. The primary objective of this study was to investigate the relationship between the iliolumbar artery and T2ELs. The secondary objective was to investigate the effect of postoperative T2ELs on long-term mortality and reintervention rates. The patients with T2ELs were screened by a color Doppler ultrasound system and PACS system. The diameter of the ilio-lumbar artery was measured within approximately 1.5 cm of its origin, and the location of its origin was recorded.

### Study population/participants

Patients with AAA who underwent EVAR in the Department of Vascular Surgery, West China Hospital, Sichuan University from June 2010 to June 2019 were enrolled. The exclusion criteria of this study were formulated as follows: (1) thoracoabdominal aortic aneurysm, para-renal abdominal aortic aneurysm, or suprarenal abdominal aortic aneurysm. (2) Patients undergoing hybrid abdominal aortic aneurysm surgery. (3) Abdominal aortic dissection aneurysm or pseudoaneurysm or perforating ulcer. (4) Ruptured abdominal aortic aneurysm or EVAR conversion to open surgery. (5) Patients who had no abdominal aortic CT before the operation and no follow-up records. (6) Patients with type I and III endoleaks.

### Data collection

The standardized electronic data system of West China Hospital, HIS system of medical records and PACS system of imaging data were used to obtain the data of the research subjects. Data were collected from patient medical records and included the following baseline and anatomical variables and operation information: age, sex, preoperative AAA diameter, neck length, maximum iliac artery diameters, anatomical characteristics of the internal iliac artery and iliolumbar artery, anesthesia method, etc. To ensure the accuracy of the data, 20% of the data were randomly checked by senior physicians in vascular surgery. If the measurement deviation was more than 10%, the senior physicians remeasured and corrected the data. Patients were divided into two groups: with or without T2ELs (The case group was T2ELs and the control group was non-T2ELs).

## Surveillance protocol

### Ethics

This study was approved by the Ethics Committee of West China Hospital, Sichuan University. All the study participants provided written informed consent stating that the clinical data could be used in clinical research.

### Analysis method

All statistical analyses were performed using SPSS version 25 (IBM Corporation, Armonk, NY). The data are presented as the mean ± standard deviation for continuous variables and as the frequency (percentage) for categorical variables, which were compared using the two-sample *t*-test, Fisher's exact test, and Pearson's *χ*^2^ test where appropriate. Overall survival and AAA-related mortality were generated using the Kaplan–Meier method, and the log-rank test was used to compare the differences. Differences with a *p* value <.05 were significant. The propensity matching score was used to calibrate the baseline.

## Result

### Baseline

A total of 603 patients were included (T2EL, 505. N-T2EL, 98, Supplementary Figure S4). Baseline characteristics are depicted in Table 1. The mean patient age was 72.0 ± 8.3 years, and males comprised 83.9% of patients. No endoleaks were identified in 505 patients (83.7%), and T2ELs were found in 98 patients (16.3%). Except for CKD, there was no significant difference between the two groups in preoperative comorbidities. T2EL patients had a lower prevalence of CKD (2.8%) than N-T2EL patients (7.1%, *p* = 0.031). Oral beta-blockers were more common in the N-T2EL group than in the T2EL group (*p* = 0.01). The median follow-up duration was 49.0 months (IQR: 42; range 1.0–136.0) in the N-T2EL group and 54.2 months (IQR: 35.5; 1.0–138.0) in the T2EL group.

**Table 1:** Baseline characteristics of patients with or without T2ELs after EVAR.

	N-T2EL (*N* = 505)	T2EL (*N* = 98)	*p*
Age	72.1 ± 8.3	72.4 ± 8.0	0.43
Male gender	426 (84.4%)	80 (81.6%)	0.502
Smoking history	309 (61.2%)	59 (60.2%)	0.855
Hypertension	342 (67.7%)	64 (65.3%)	0.641
Diabetes	66 (13.1%)	12 (12.2%)	0.824
COPD	104 (20.6%)	24 (24.5%)	0.388
Dyslipidemia	27 (27%)	7 (7.1%)	0.483
Coronary artery disease	92 (18.2%)	22 (22.4%)	0.328
Chronic kidney disease	14 (2.8%)	7 (7.1%)	0.031
Anesthesia method			0.377
Local anesthesia	370 (73.3%)	76 (77.6%)	
General anesthesia	135 (26.7%)	22 (22.4%)	
Medication			
Statin	90 (17.8%)	20 (20.4%)	0.544
Antiplatelet	413 (81.8%)	84(85.7%)	0.272

Data are presented as *n* (%) or mean ± standard deviation.

COPD, chronic obstructive pulmonary disease; ACEI,  angiotensin-converting enzyme inhibitor.

The vascular morphologic characteristics are shown in Table 2. Our results showed that the proportion of thrombus in the aneurysm cavity (*p* = 0.011), the diameter of the median sacral artery (*p* = 0.04), lumbar artery (*p* = 0.015), inferior mesenteric artery (*p* < 0.001) and the number of lumbar arteries (*p* = 0.019) were significantly different between the two groups. The diameter of the ILA in the N-T2EL group was 2.3 ± 0.7 mm, and in the T2EL group, the right ILA was 2.4 ± 0.6 mm, and the left ILA was 2.5 ± 1.4 mm (Table 3).

**Table 2:** Univariable analysis of patients with or without T2ELs after EVAR.

	OR (95% CI)	*p*	OR (95% CI)	*p*
Age	0.990 (0.964–1.016)	0.429	0.993 (0.967–1.020)	0.616
Max-diameter of AAA (mm)	1.009 (0.993–1.025)	0.263	1.004 (0.988–1.021)	0.606
diameter of AAA neck (mm)	1.048 (0.969–1.133)	0.244	1.035 (0.95–1.128)	0.427
Length of AAA neck (mm)	1.000 (0.983–1.017)	0.965	0.998 (0.980–1.016)	0.804
Proportion of thrombus	0.294 (0.113–0.763)	0.012	0.72 (0.262–1.977)	0.523
*α* angle	1.003 (0.996–1.011)	0.409	1.00 (0.992–1.009)	0.908
*β* angle	1.002 (0.994–1.009)	0.671	1.00 (0.992–1.008)	0.994
Diameter of right CIA	1.007 (0.985–1.030)	0.515	1.008 (0.985–1.032)	0.513
Diameter of left CIA	1.015 (0.990–1.040)	0.244	1.020 (0.999–1.042)	0.068
Diameter of right IIA	1.017 (0.983–1.052)	0.324	1.016 (0.981–1.053)	0.374
Diameter of left IIA	1.020 (0.993–1.048)	0.148	1.021 (0.991–1.052)	0.168
Diameter of MSA	1.284 (1.011–1.631)	0.040	0.962 (0.746–1.242)	0.767
Diameter of LA	1.520 (1.067–2.164)	0.02	1.161 (0.775–1.740)	0.468
Diameter of IMA	1.056 (1.241–1.829)	<0.001	1.137 (0.919–1.407)	0.236
Numbers of patent LA	1.390 (1.195–1.618)	<0.001	1.100 (0.927–1.306)	0.273
Diameter of right ILA	1.394 (0.996–1.951)	0.053	1.164 (0.810–1.673)	0.412
Diameter of left ILA	1.221 (0.960–1.553)	0.103	1.101 (0.865–1.401)	0.433

HR, odds ratio; CIA, celiac internal artery; MSA, median sacral artery. IMA, inferior mesentery artery; IIA, internal iliac artery; LA, lumbar artery; ILA, ilioiliac lumbar artery.

**Table 3:** Anatomic characteristics of patients.

	T2EL (−)	N-T2EL (+)	*p*
Max-diameter of AAA (mm)	53.7 ± 13.4	55.3 ± 13.8	0.263
Diameter of neck	21.3 ± 2.6	21.6 ± 2.9	0.244
Length of neck	28.1 ± 12.6	28.1 ± 13.1	0.965
Proportion of thrombus	0.3 (0.1, 0.5)	0.2 (0.1, 0.4)	0.011
*α* angle	32.5 ± 27.3	35.1 ± 30.5	0.410
*β* angle	48.2 ± 29.8	49.6 ± 30.6	0.671
Diameter of right CIA	18.9 ± 9.2	19.5 ± 9.7	0.515
Diameter of left CIA	17.3 ± 7.6	18.3 ± 10.1	0.242
Diameter of right IIA	10.9 ± 5.9	11.6 ± 5.5	0.332
Diameter of left IIA	11.2 ± 6.7	12.3 ± 8.1	0.143
Diameter of MSA	0.9 ± 0.9	1.1 ± 0.9	0.040
Diameter of LA	2.5 ± 0.6	2.6 ± 0.6	0.015
Diameter of IMA	2.3 ± 2.5	2.7 ± 1.2	<0.001
Complicated with CIA	125 (24.8)	28 (28.6)	0.427
Complicated with IIA	78 (15.4)	10 (10.2)	0.179
Number of patent LA	6.0 (4.0, 6.0)	6.0 (6.0, 7.0)	0.019
Diameter of right ILA	2.3 ± 0.7	2.4 ± 0.6	0.052
Diameter of Left ILA	2.3 ± 0.7	2.5 ± 1.4	0.068

Data are presented as *n* (%) or mean ± standard deviation.

CIA, celiac internal artery; MSA, median sacral artery; IMA, inferior mesentery artery; IIA, internal iliac artery; LA, lumbar artery; ILA, ilioiliac lumbar artery.

In Table 4, patient characteristics were compared based on the type of IIA embolization performed and primary IIA occlusion. A total of 443 individuals did not undergo any preoperative IIA intervention (T2EL, 72.4%. N-T2EL, 73.7%). In addition, 160 patients (26.5%) received the intervention. Sixty patients (T2EL, 10.7%. N-T2EL, 6.1%) accepted unilateral stent-covered IIA without embolization, and 10 patients (T2EL, 1.8%. N-T2EL, 1.0%) bilateral stent coverage without embolization. Unilateral stent coverage with embolization was performed in 59 patients (T2EL, 9.5%. N-T2EL, 11.2%), and bilateral stent coverage with embolization was completed in 31 patients (T2EL, 4.4%). N-T2EL, 9.2%). Our analysis showed no significant difference between the two groups in the intervention mode of IIA (*p* = 0.224). There was also no difference in primary iliac artery occlusion between the two groups (*p* = 0.723). No variable was significantly different in the anatomic origin of ILA between the two groups.

**Table 4:** Characteristics of the internal iliac artery and ILA.

	T2EL (−)	T2EL (+)	*p*
Internal iliac intervention			0.224
No intervention	372 (73.7%)	71 (72.4%)	
Unilateral stent coverage	54 (10.7%)	6 (6.1%)	
Bilateral stent coverage	9 (1.8%)	1 (1.0%)	
Unilateral stent coverage with embolization	48 (9.5%)	11 (11.2%)	
Bilateral stent coverage with embolization	22 (4.4%)	9 (9.2%)	
Primary internal iliac occlusion			0.723
No occlusion	473 (93.7%)	94 (95.9%)	
Unilateral occlusion	25 (5.0%)	4 (4.1%)	
Bilateral occlusion	7 (1.4%)	0 (0%)	
Left origin of ilio-lumbar artery			0.26
Posterior division of IIA	167 (33.1%)	41 (41.8%)	
The trunk of IIA	313 (62.0%)	51 (52.0%)	
Left CIA	14 (2.8%)	4 (4.1%)	
Right origin of ilio-lumbar artery			0.223
Posterior division of IIA	109 (21.6%)	15 (15.3%)	
The trunk of IIA	385 (76.2%)	79 (80.6%)	
Right CIA	4(0.8%)	2(2.0%)	

Data are presented as *n* (%).

In the univariate analysis (Table 2), our results showed that T2ELs may have larger MSA (OR = 1.284, *p* = 0.04), LAs (OR = 1.520, *p* = 0.015), and IMA (OR = 1.056, *p* < 0.001) and more LAs (OR = 1.390, *p* = 0.019). And the patients with T2EL may have smaller proportion of thrombus in the aneurysm cavity (OR = 0.294, *p* = 0.012).

The factors with *p* < 0.1 in the univariate analysis in Tables 1, 2 were subjected to a subsequent multivariate analysis to evaluate the association with T2ELs. In Table 5, our multivariate analysis showed the number of LAs (before, OR: 1.351, 95% CI: 1.144–1.597, *p* < .001. After, OR: 1.349, 95% CI: 1.140–1.595, *p* < .001), and diameter of IMA (Before, OR: 1.330, 95% CI: 1.08–1.637, *p* = .007. After, OR: 1.328, 95% CI: 1.078–1.636, *p* = .008) were identified to be significantly associated with T2Els, which was consistent after adjusting for the IIA.

**Table 5:** Multivariate analysis before (M1) and after (M2) adjusting for primary hypogastric artery condition.

	Before		After		PSM	
	OR (95% CI)	*p*	OR (95% CI)	*p*	OR (95% CI)	*p*
CKD	2.596 (0.983–6.855)	0.054	2.612 (0.986–6.920)	.053	0.383 (0.145–1.015)	0.053
Diameter of MSA	1.102 (0.854–1.422)	0.497	1.095 (0.847–1.415)	.489	1.095 (0.847–1.415)	0.489
Diameter of IMA	1.330 (1.08–1.637)	.007	1.328 (1.078–1.636)	.008	1.328 (1.078–1.636)	0.008
Numbers of patent LA	1.351 (1.144–1.597)	<.001	1.349 (1.140–1.595)	<.001	1.349 (1.140–1.595)	<.001
Proportion of thrombus	0.423 (0.157–1.179)	.100	0.424 (0.152–1.182)	.101	0.424 (0.152–1.182)	0.101
Diameter of right ILA	1.389 (0.957–2.017)	.084	1.394 (0.958–2.028)	.083	1.394 (0.958–2.028)	0.083
Primary internal iliac occlusion						
Unilateral occlusion	—	—	0.821 (0.227–2.976)	.764	1.606 (0.146–17.641)	0.698
Bilateral occlusion	—	—	0.623 (0.057–6.840)	.698	1.319 (0.143–12.128)	0.807
Internal iliac intervention						
Unilateral stent coverage	0.569 (0.230–1.407)	.222	0.706 (0.155–3.224)	.653	0.281 (0.023–3.460)	0.321
Bilateral stent coverage	0.644 (0.076–5.435)	.686	1.023 (0.042–24.696)	.989	0.198 (0.019–2.032)	0.173
Unilateral stent coverage with embolization	1.093 (0.520–2.301)	.814	1.324 (0.314–5.575)	.702	0.287 (0.030–2.773)	0.281
Bilateral stent coverage with embolization	2.247 (0.957–5.277)	.063	3.564 (0.289–43.961)	.083	0.372 (0.033–4.237)	0.425

CIA, celiac internal artery; MSA, median sacral artery; IMA, inferior mesentery artery; IIA, internal iliac artery; LA, lumbar artery; ILA, ilioiliac lumbar artery.

**Figure 1:**
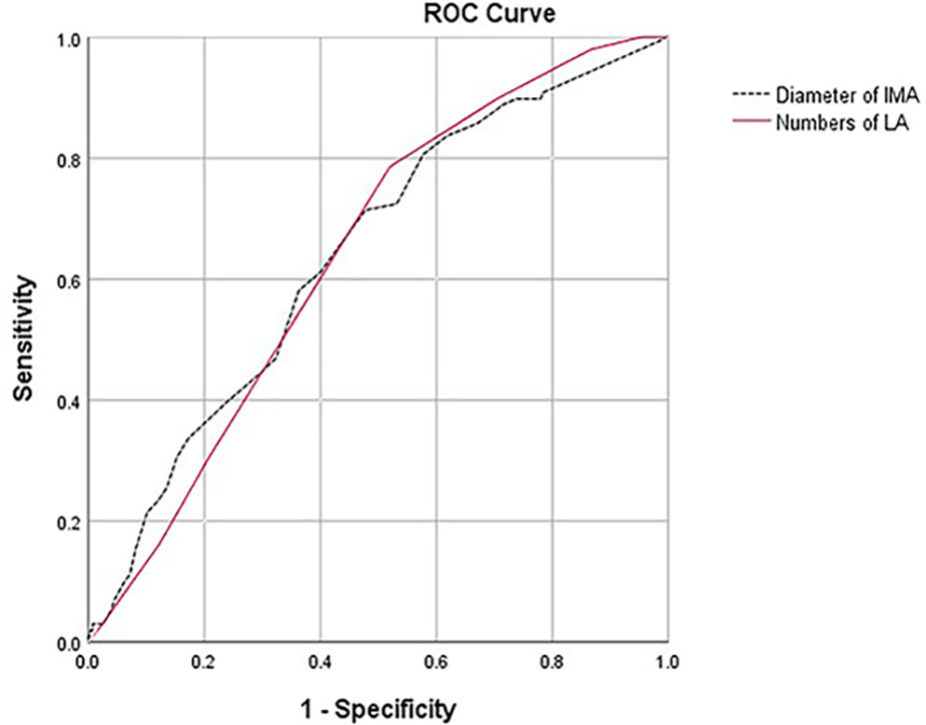
ROC analysis showed that there was no difference in predictive value between the number of LAs (AUC = 0.640, 95% CI: 0.587–0.693, sensitivity = 0.714, specificity = 0.522) and the diameter of the IMA (AUC = 0.642, 95% CI: 0.584–0.699, sensitivity = 0.786, specificity = 0.48, *p* = 0.972).

ROC analysis (Figure 1) showed that the cutoff value for the number of LAs was 6 (AUC = 0.640, 95% CI: 0.587–0.693, sensitivity = 0.714, specificity = 0.522) and for the diameter of the IMA was 2.5 mm (AUC = 0.642, 95% CI: 0.584–0.699, sensitivity = 0.786, specificity = 0.48).

**Figure 2:**
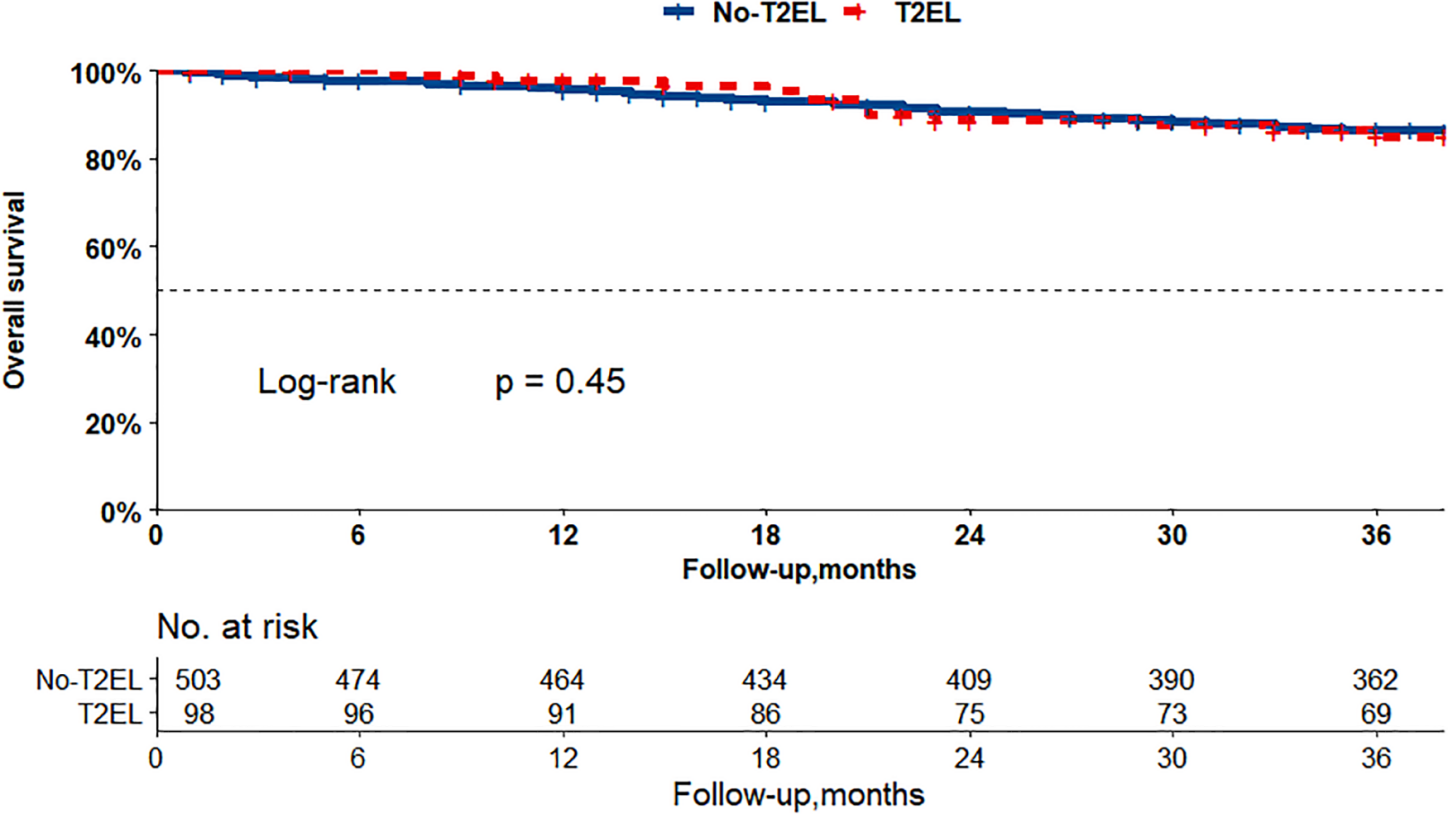
Overall survival did not differ between the patients with T2ELs and those without T2ELs. (*p* = 0.45, log rank test).

**Figure 3:**
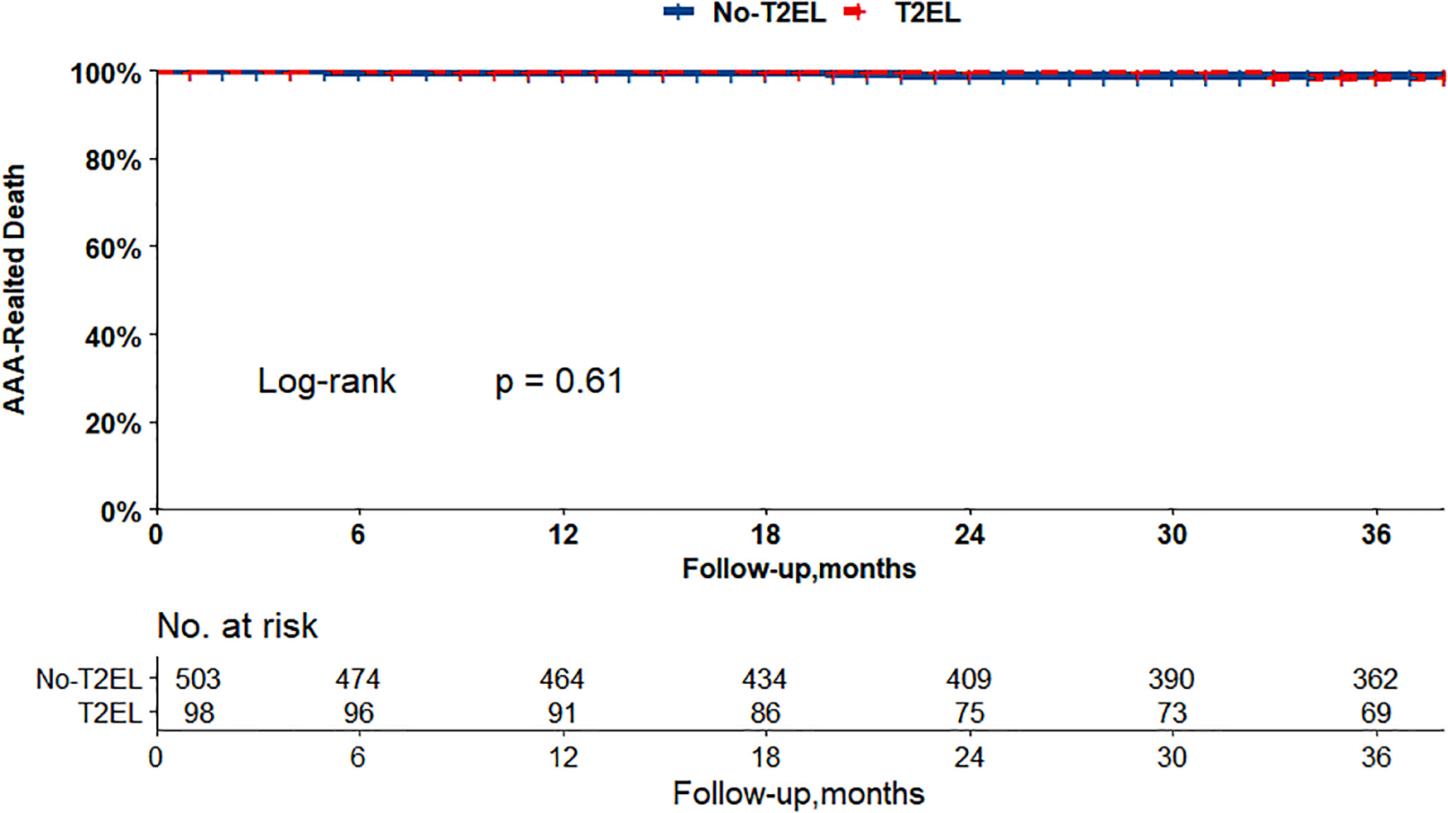
AAA-related survival did not differ between the patients with T2ELs and those without T2ELs. (*p* = 0.61, log rank test).

**Figure 4:**
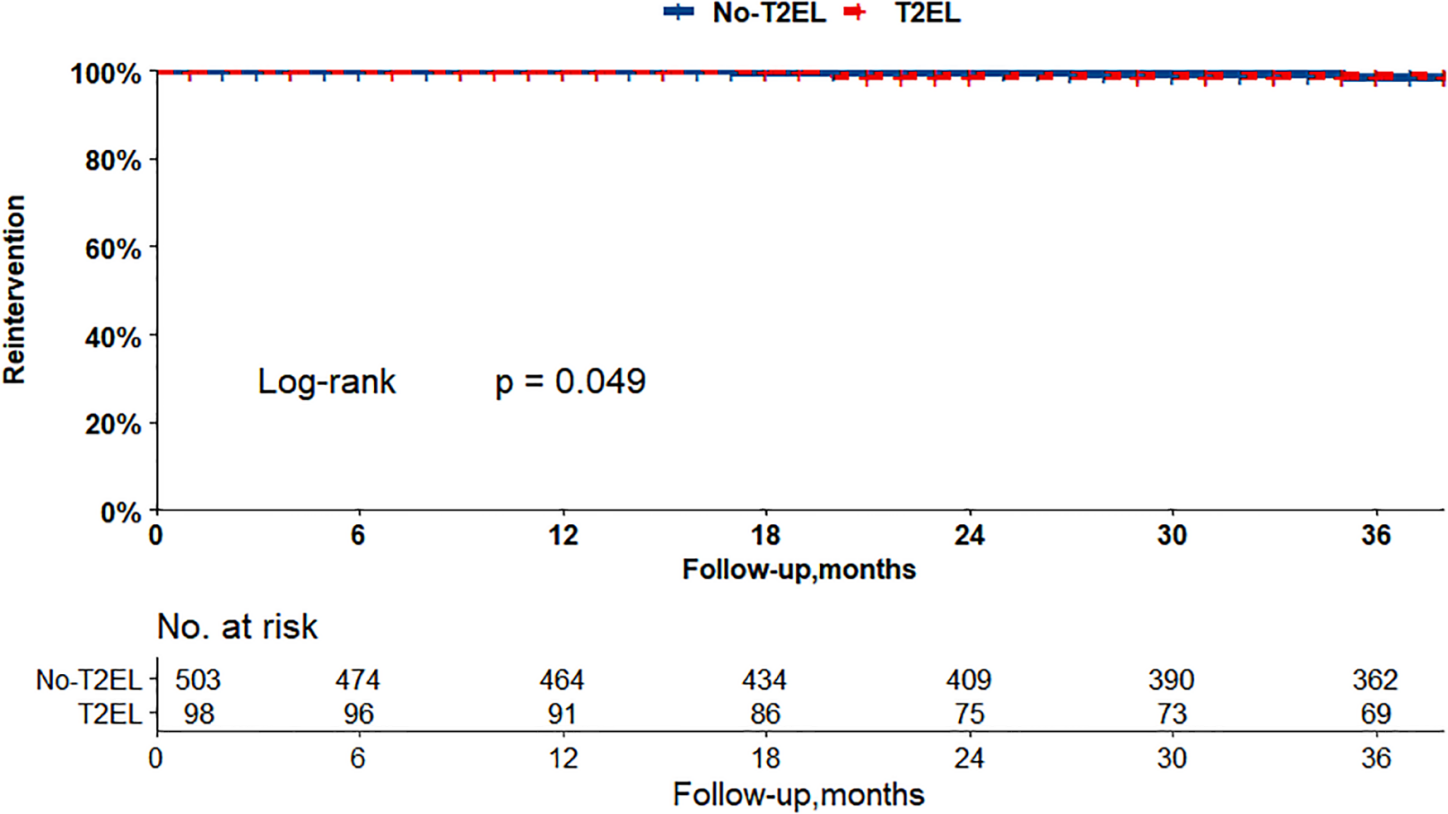
Patients with T2ELs had more reinterventions than those without T2ELs (*p* = 0.049, log rank test).

The Kaplan–Meier curves in Figures 2, 3 show that there were no differences between the T2EL group and N-T2EL group in overall survival (T2EL, 71.9%, N-T2EL, 59.9% at 8 years, *p* = 0.45) and freedom from AAA-related death (T2EL, 95%, N-T2EL, 91.3% at 8 years, *p* = 0.61). The reintervention-free survival rates at 8 years were 96.1% and 77.3% in patients with and without T2ELs, respectively, which were significantly different between the two groups (*p* = 0.049, Figure 4).

Variables of PSM included all anatomical data, age, sex, and preoperative comorbidities (Table 6, univariable analysis after PSM). The propensity score matching (PSM) included 391 patients (T2ELs vs. non-T2ELs: 91 vs. 297), and the multivariate analysis after PSM in Table 5 indicated that the independent risk factors for T2ELs were still the number of LAs (OR: 1.349, 95% CI: 1.140–1.595, *p* < 0.001) and the diameter of the IMA (OR: 1.328, 95% CI: 1.078–1.636, *p* = 0.008). There was no significant difference between the intervention modes of IIA and ILA in the two groups. Additionally, the Kaplan–Meier curves after PSM found that T2ELs group had a higher rate of reintervention (*p* = 0.034) in Supplementary Figure S3, but there was no difference in overall survival and AAA-related death between the two groups (Supplementary Figures S1 and S2).

**Table 6:** Univariable analysis of patients with or without T2EL after EVAR after PSM.

	Univariable analysis Hazard ratio (95% CI)	*p*
Age	0.993 (0.967–1.020)	0.616
Max-diameter of AAA (mm)	1.004 (0.988–1.021)	0.606
diameter of AAA neck (mm)	1.035 (0.95–1.128)	0.427
Length of AAA neck (mm)	0.998 (0.980–1.016)	0.804
Proportion of thrombus	0.72 (0.262–1.977)	0.523
α angle	1.00 (0.992–1.009)	0.908
β angle	1.00 (0.992–1.008)	0.994
Diameter of right CIA	1.008 (0.985–1.032)	0.513
Diameter of left CIA	1.020 (0.999–1.042)	0.068
Diameter of right IIA	1.016 (0.981–1.053)	0.374
Diameter of left IIA	1.021 (0.991–1.052)	0.168
Diameter of MSA	0.962 (0.746–1.242)	0.767
Diameter of LA	1.161 (0.775–1.740)	0.468
Diameter of IMA	1.137 (0.919–1.407)	0.236
Numbers of patent LA	1.100 (0.927–1.306)	0.273
Diameter of right ILA	1.164 (0.810–1.673)	0.412
Diameter of left ILA	1.101 (0.865–1.401)	0.433

CIA, celiac internal artery. MSA, median sacral artery. IMA, inferior mesentery artery. IIA, internal iliac artery. LA, lumbar artery. ILA, ilio-iliac lumbar artery.

## Discussion

The management of T2EL remains controversial in the current literature, and it has several definitions, including “early”, “late”, “persistent” and “isolated” type II endoleaks. T2ELs were observed in 10.2% of patients after EVAR (9), and 30% to 50% of these resolved spontaneously (9, 26). In a Japanese nationwide analysis, persistent T2EL was defined as T2EL detected after EVAR on initial contrast-enhanced CT and during follow-up or new T2EL not documented at the end of EVAR but reported at any point during follow-up (27). A correlation between persistent T2ELs (p-T2ELs) and late adverse events, including aneurysm sac enlargement, reintervention, rupture, and abdominal aortic aneurysm-related mortality after endovascular aneurysm repair, was demonstrated. In addition to p-T2ELs, older age, female sex, chronic kidney disease, and dilated proximal neck were associated with sac enlargement.

Wang et al. (28) analyzed 10-year follow-up results and found that T2ELs were significantly associated with aneurysm sac growth, but no association with survival was observed. The low overall survival rate in our analysis may be related to COVID-19.

Current guidelines, such as the 2019 European Society for Vascular Surgery (ESVS) guideline and Society for Vascular Surgery implementation of clinical practice guidelines, have recommended conservative management, and intervention was indicated for significant sac expansion (≥10 mm or 5 mm) (1, 29). Although reintervention for T2ELs after EVAR could achieve some clinical effects (30–32), a study (33) found that embolization procedures were generally ineffective in preventing further expansion of abdominal aortic aneurysms in patients with T2ELs after EVAR. The risk of repeated intervention after reintervention for T2ELs exists, and the key to treating T2ELs has shifted from reintervention to prevention.

Therefore, identifying risk factors for T2ELs and early intervention in high-risk patients are key to treatment. Our study found that the IMA diameter and the number of LAs were independent risk factors, which was consistent with most studies (8, 13, 22, 34). Through ROC curve analysis, this study determined the number of lumbar arteries (≥6) and the cutoff of IMA diameter (≥2.5 mm). Some studies have identified IMA ≥3 mm as a risk factor for T2ELs (11, 12).

IIA embolization has also been suggested as a risk factor for T2ELs in some studies (35, 36). They thought IIA embolization was more likely to increase the redistribution of blood flow from the lumbar arteries and IMA branches than IIA stent coverage alone. The formation of collateral circulation may also be associated with ILA. Meishi et al. (22) thought that the iliolumbar artery arising from the IIA was a major source of T2ELs. In their study, no significant impact of IIA embolization on T2ELs was observed after analyzing 375 patients. Of all 603 patients in our article, 9.8% received unilateral and 5.1% bilateral IIA embolization. The multivariate analysis showed that the different interventions for IIA were not associated with T2ELs, regardless of the IIA status. Currently, iliac branch devices are used to preserve at least one IIA. From existing studies (37, 38) and the limited evidence presented in our article, preservation of the IIA does not appear to increase the incidence of T2ELs (22).

**Figure 5:**
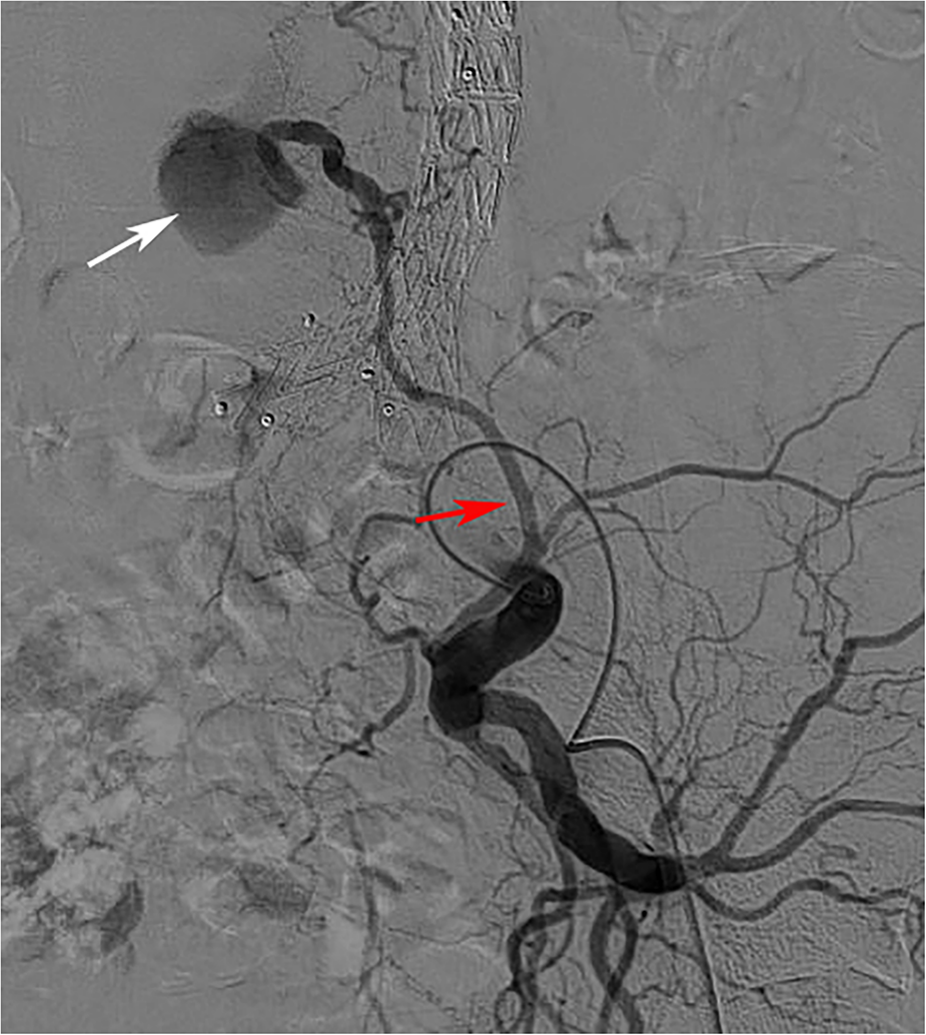
The iliolumbar artery was a source of type 2 endoleak. (Red arrow is the ILA, white arrow is the aneurysm cavity).

In addition, the relationship between ILA and T2ELs has not been compared, although it has sometimes been found to be the source of T2ELs during follow-up (Figure 5). In our study, the right ILA diameter measured based on abdominal CTA was 2.31 ± 0.65 mm, and the left ILA was 2.30 ± 0.66 mm, which was similar to previous results based on human anatomy, which reported that the diameter was 2.7 ± 0.6 mm (39). We found that 28.0% of ILAs originated from the posterior of the IIA, 69.9% from the main trunk of the IIA, and 0.2% from the CIA. Kiray et al. (40) reported an average ILA diameter of 3.7 ± 0.7 mm, and Teli et al. (25) reported an average ILA diameter of 3.5 ± 0.5 mm. Koc et al. (23) reported that the ILA diameter originating from the main trunk of the IIA was smaller than that originating from the posterior branch of the IIA. In addition, the iliac lumbar artery mainly originates from the main internal iliac artery and less from the posterior branch of the IIA and CIA (25, 39, 40). This finding was similar to our study.

In the T2ELs caused by ILA, we found that ILA tended to be backward and upward, but only 2% of T2ELs were caused by ILA. There was no significant difference in the anatomical characteristics of the ILA in univariate analysis, and the right ILA diameter also showed no difference in multivariate analysis (*p* = 0.83). T2ELs from the IIA reported in the past are relatively rare, and the incidence of most previous report series is between 0% and 3.8% (21, 41), and no correlation was demonstrated in previous studies (22, 42). Other risk factors associated with T2ELs include chronic kidney disease, advanced age, aneurysm sac volume, and aneurysm sac thrombus volume (34, 43–46). Although our study did not find a statistical correlation between ILA and T2ELs, T2ELs caused by ILA still deserve attention.

Pre-embolization for the IMA or aneurysm sac in high-risk patients seems to be of greater benefit (14, 47, 48), and it could suppress aneurysm sac expansion and reduce the reintervention rate. A network meta-analysis (49) suggested that IMA embolization demonstrated benefits in achieving long-term aneurysm sac stability and lowering the risk of secondary surgery. Nonselective embolization of aneurysm sac side branches more effectively reduces the incidence of T2ELs, while IMA embolization alone or in combination with aneurysm sac coil embolization enhances the clinical benefits of EVAR. Sun et al. (49) analyzed whether nonselective preemptive aneurysm sac embolization could prevent T2ELs in the short and mid-term, and they interestingly found that the IMA diameter showed continuous regression in the embolization group.

However, Kontopodis et al. (50) in their meta-analysis that included four random studies, suggested that preemptive embolization confers no clinical benefits in EVAR, but their data were limited and had low certainty. Additionally, Väärämäki et al. (51) found that the strategy of routinely embolizing the IMA does not seem to yield any significant clinical benefit and should therefore be abandoned. Therefore, more standardized, and high-quality studies are needed to explore the therapeutic effects of preemptive embolization.

There are several other limitations that should be noted. The present study excluded patients with type I and III endoleaks at any time during follow-up. This may reduce the number of patients with T2ELs because some patients with T2ELs may also have type I or type III endoleaks. Another limitation is the definition of T2EL. Previous reports use various terms, such as “early”, “late”, and “persistent” T2ELs. However, we did not classify T2ELs in this study. On the one hand, there was recall bias during the telephone follow-up; on the other hand, some patients did not have regular follow-up after surgery, so the occurrence time of endoleaks was not clear.

Finally, the mode of IIA embolization was not a routine procedure and was only performed in a relatively specialized anatomical population. Aortoiliac aneurysms with insufficient distal anchor area of the common iliac artery may lead to type Ib endoleaks (52). In some aortoiliac abdominal aortic aneurysms or AAA with internal iliac aneurysms and with a short common iliac artery, stents need to extend to the external iliac artery or even embolize the ipsilateral internal iliac artery. Future studies may need to collect the anatomical characteristics of the patent lateral branches after EVAR to identify the risk factors further accurately for type II endoleaks through the changes before and after surgery.

## Conclusion

The iliolumbar artery and the different internal iliac artery interventions may not increase the incidence of T2Els. But the number of patent lumbar arteries (≥6) and the diameter of the inferior mesenteric artery (≥2.5 mm) were independent risk factors for T2ELs. More rigorous studies are still needed to explore the risk factors for T2ELs.T2ELs was associated with the reintervention but did not affect long-term survival or increase aneurysm-related mortality after EVAR.

## Data Availability

The original contributions presented in the study are included in the article/**Supplementary Material**, further inquiries can be directed to the corresponding authors.

## Supplementary Material

SUPPLEMENTAL FIGURE 1

Overall survival did not differ between T2ELs group and non-T2Els group after PSM (*p* = 0.58, log rank test).

SUPPLEMENTAL FIGURE 2

AAA-related survival did not differ between T2ELs group and non-T2Els group after PSM (*p* = 0.6, log rank test).

SUPPLEMENTAL FIGURE 3

Patients with T2ELs had more reinterventions than those without T2ELs. (*p* = 0.034, log rank test).

SUPPLEMENTAL FIGURE 4.

Flow chart of cases inclusion and expulsion.
