# Supplementary material for: Patent iliolumbar artery increase no risk of type II endoleaks after endovascular abdominal aortic aneurysm: a case-control study
Source: Front Cardiovasc Med. 2023 Aug 11;10:1210248. doi: 10.3389/fcvm.2023.1210248 (PMC10455956; doi:10.3389/fcvm.2023.1210248)
Supplement: Supplementary file 1 [file Datasheet1.docx]

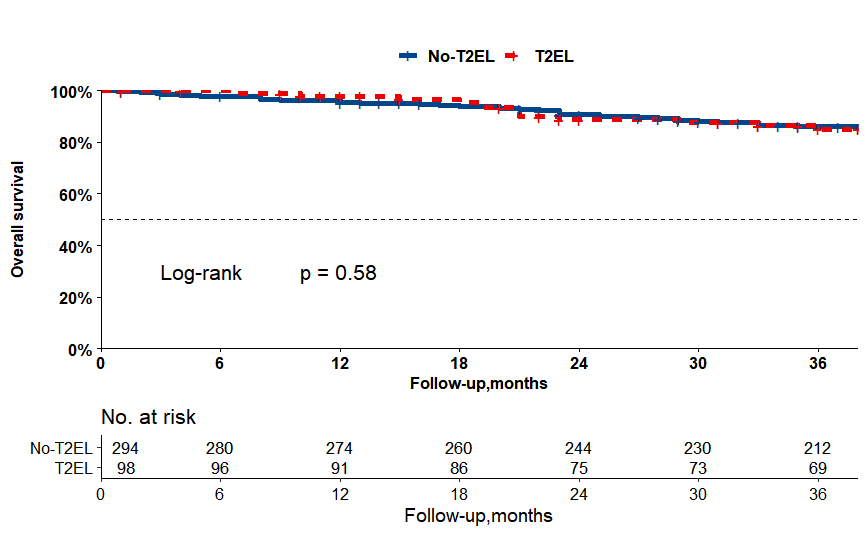


Supplementary Figure 1. *Overall survival did not differ between T2ELs group and non-T2Els group after PSM. (p = 0.58, log rank test).*


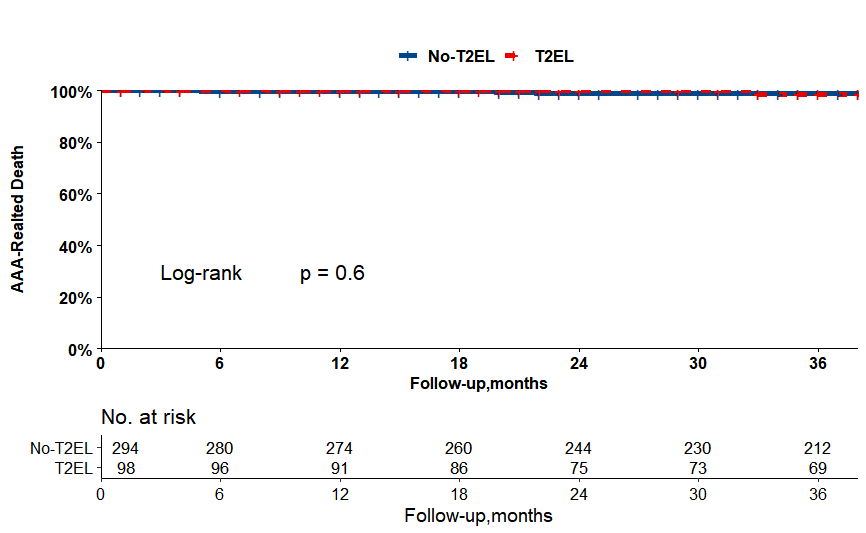


Supplementary Figure 2. *AAA-related survival did not differ between T2ELs group and non-T2Els group after PSM.. (p = 0.6, log rank test).*


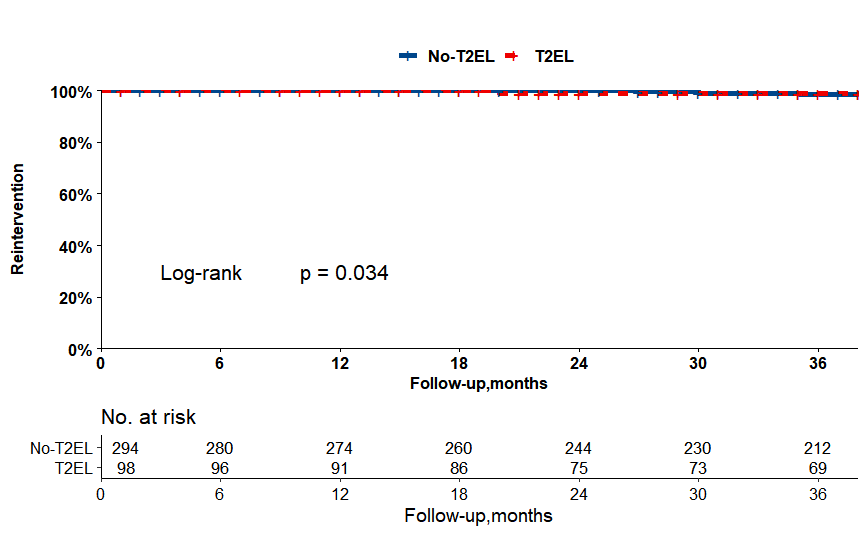


Supplementary Figure 3. *Patients with T2ELs had more reinterventions than those without T2ELs. (p = 0.034, log rank test).*


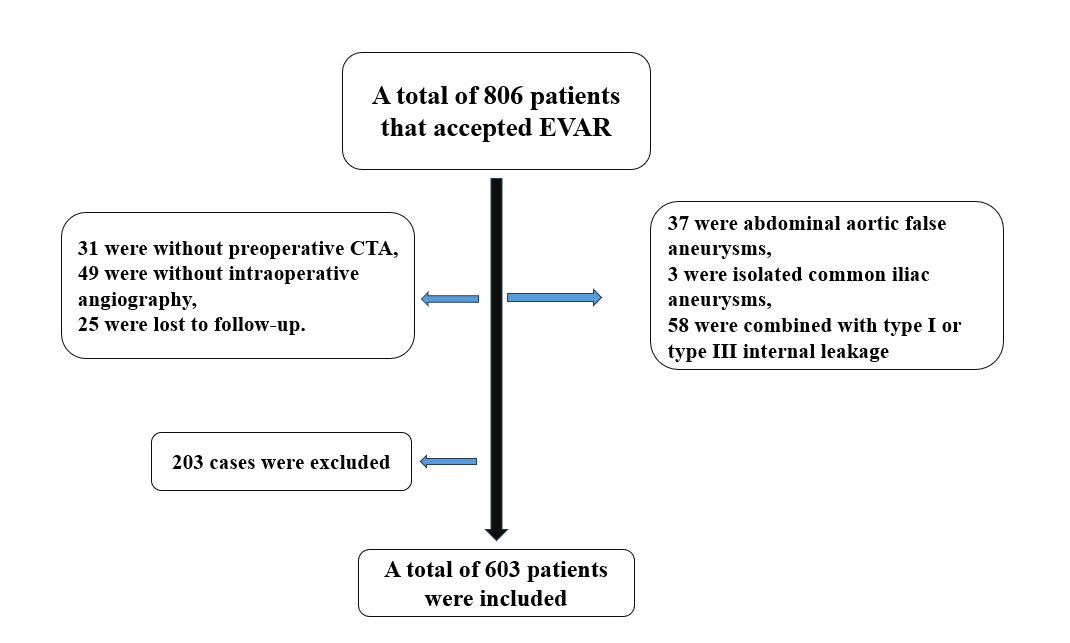


Supplementary Figure 4*. Flow chart of cases inclusion and expulsion*
